# Insights into the *p*-nitrophenol adsorption by amidoxime-modified poly(acrylonitrile-*co*-acrylic acid): characterization, kinetics, isotherm, thermodynamic, regeneration and mechanism study

**DOI:** 10.1039/d0ra10910j

**Published:** 2021-02-19

**Authors:** Shihab Ezzuldin M. Saber, Siti Nurul Ain Md Jamil, Luqman Chuah Abdullah, Thomas Shean Yaw Choong, Teo Ming Ting

**Affiliations:** Department of Chemical and Environmental Engineering, Faculty of Engineering, Universiti Putra Malaysia UPM Serdang 43400 Selangor Malaysia shihab.ezzuldeen@gmail.com; Department of Chemistry, Faculty of Science, Universiti Putra Malaysia UPM Serdang 43400 Selangor Malaysia ctnurulain@upm.edu.my; Centre of Foundation Studies for Agricultural Science, Universiti Putra Malaysia UPM Serdang 43400 Selangor Malaysia; Institute of Tropical Forestry and Forest Products (INTROP), Universiti Putra Malaysia UPM Serdang 43400 Malaysia; Radiation Technology Division, Malaysian Nuclear Agency 43000 Kajang Selangor Malaysia; North Refineries Company, Ministry of Oil of Iraq Baiji Salahuddin Iraq

## Abstract

This study performs an appraisal of the adsorptive capacity of amidoxime-modified poly(acrylonitrile-*co*-acrylic acid) or abbreviated as (AO-modified poly(AN-*co*-AA)) for the *p*-nitrophenol (PNP) adsorption, from aquatic environments *via* batch system. The AO-modified poly(AN-*co*-AA) polymer was developed with redox polymerization, and then altered by using hydroxylamine hydrochloride (HH). Tools used to describe the physicochemical and morphological characteristics of the AO-modified poly(AN-*co*-AA) were Fourier transform infrared (FTIR) spectroscopy, CHN elemental analysis, X-ray diffraction analysis (XRD) and scanning electron microscopy (SEM). The adsorption kinetics were examined by pseudo-first order, pseudo-second order, Elovich and intraparticle diffusion kinetic models. Meanwhile, the isotherms were investigated by Langmuir, Freundlich, Temkin and Redlich–Peterson models. It was found that the adsorption was best fitted with pseudo-second order, and agreed with both Langmuir and Freundlich isotherm models. It was described best with the Freundlich isotherm due to highest *R*^2^ (0.999). The maximum adsorption capacity was 143.06 mg g^−1^ at 298 K, and thermodynamic functions showed that the adsorption process was exothermic. Also, following five regeneration cycles, the adsorbent recorded 71.7% regeneration efficiency. The finding in this study indicates that the AO-modified poly(AN-*co*-AA) is an effective adsorbent to remove PNP from an aqueous solution.

## Abstract 2

This study performs an appraisal of the adsorptive capacity of amidoxime-modified poly(acrylonitrile-*co*-acrylic acid) for the *p*-nitrophenol (PNP) adsorption, from aqueous solutions.

## Graphical abstract

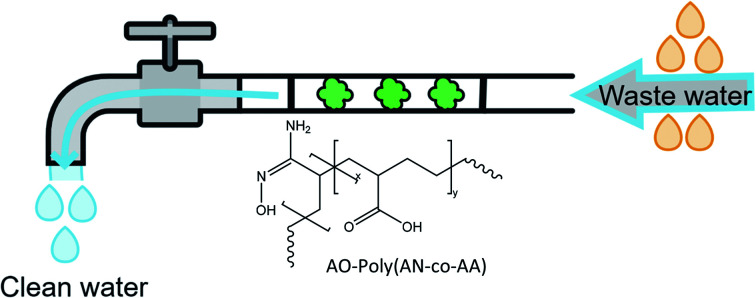

## 1. Introduction

Researchers and environmentalists are highly concerned about the prevalence of phenol and its compounds in the environment. The presence of phenol pollutants in water causes unpleasant smells, poor taste and toxicity across all concentrations.^1^ The manufacturing of dyes, paints, explosives, herbicides and pesticides, and feedstock involve the utilization of phenol compounds as solvents and precursors, across a wide range of chemical and pharmaceutical sectors. In turn, these operations are the cause of the rising level of phenolic compound discharge, particularly with *p*-nitrophenol (PNP) bearing effluents into ecosystems. Wastewater, which includes PNP, has a negative impact on water bodies it encounters, producing significant dangers for the local flora and fauna, along with public health.^2,3^ It was reported that liver and kidney damage, tissue erosion, paralysis of the central nervous system, headache and dizziness are all possible symptoms due to phenolic compound effects.^4^ Phenolic compounds have been listed as priority contaminants by the US Environmental Protection Agency (EPA) because of their toxicity at low concentrations. The sill concentration of phenols in water should not be higher than 2 μg L^−1^, as specified by the US (EPA), and 1.0 μg L^−1^ according to the recommendation of the World Health Organization (WHO).^5,6^ Therefore, reducing the concentration of phenol components from industrial wastewater in order to adhere to discharge regulations is a topic that requires attention.

There have been a number of methods used to purify phenols-containing wastewaters, such as chemical precipitation/oxidation,^7^ biodegradation,^8^ membrane separation,^9^ reverse osmosis^10^ and adsorption.^11,12^ Of these approaches, PNP sequestration was achieved most effectively through adsorption because of the simple design involved, together with its flexibility across a large number of pollutants and straightforward application.^13,14^ For the purpose of removing different pollutants, including biomass waste,^15^ zirconium silicate,^16^ nanographite oxide,^2^ zeolite and bentonite,^17^ and polymeric adsorbents,^18^ a wide range of adsorbents have been examined. Of these adsorbents, polymeric materials showed the greatest efficiency, as the polymers can be manipulated through the insertion of specific active functional groups based on the targeted pollutants and synthesized in favourable environments. Furthermore, polymeric materials can be used across multiple adsorption cycles. In turn, the creation of innovative facile adsorbents with straightforward preparation routes, low cost, high efficiency and high regeneration ability is an important priority.

For the purposes of wastewater treatment, industrial dye treatment, heavy metal treatment, polymeric adsorbents and its derivatives (*e.g.*, polyurea, polythiophenes and polyacrylonitrile (PAN)) have been developed.^19^ Notably, a number of contemporary studies included the functionalization or surface modifications of these polymers, for the purpose of boosting the effectiveness and selectivity of specific pollutants. An example of this is where El-Aassar *et al.* (2016) synthesized poly(acrylonitrile-*co*-styrene) and its surface functionalized with carboxylic acid groups for dye decolorization.^20^ Furthermore, the synthesis and functionalization of poly(vinylbenzyl chloride) beads with an amidoxime group for the high adsorption of dyes and heavy metals were also described.^21^

Due to its notable thermal stability and significant levels of mechanical strength, polyacrylonitrile (PAN) is employed in the adsorption process.^22^ The PAN-based polymer was employed as an adsorbent due to the presence of the reactive cyano group along the adsorbent chains that can be chemically modified into active amidoxime groups to capture the target analyte (PNP). For use in specific circumstances, including environmental and medical industrial applications, a number of monomers (including methacrylic acid and itaconic acid) have been copolymerized with acrylonitrile (AN).^23^ Sahiner and Ilgin produced amphiphilic, pH and magnetic field sensitive polymeric particles through altering the poly(acrylonitrile-*co*-4-vinylpyridine) (p(AN-*co*-4-VP)) core–shell nanoparticles.^88^ In the work of Yang *et al.* (2017), the production of a thin-film nanofibrous composite ultrafiltration (UF) membrane consisting of ultrathin poly(acrylonitrile-*co*-acrylic acid) was described.^89^ Along the same lines, Adeyi *et al.* (2019b) synthesized thiourea functionalized poly(acrylonitrile-*co*-acrylic acid) *via* free radical polymerization in order to treat cationic dyes from aqueous solution.^24^

The synthesis of the polyacrylonitrile based copolymer and terpolymer *via* redox method has been described before. The polymerization applied convenient methods, in which it involved polymerization at low temperatures (45 °C), at shorter times (up to 3 hour), and was carried out by using deionized water as the reaction medium.^25,26^ The yield of polymerization was reported to be as high as 77% for the copolymerization of acrylonitrile with acrylamide,^27^ and 71% for the terpolymerization of acrylonitrile with acrylate monomer and fumaronitrile.^25^ This method is considered as an environmentally friendly method due to the lower utilization of organic solvents.

For the current study, acrylic acid (AA) was chosen as a monomer. This involved carboxylic acid with a double bond, which was used to integrate a carboxyl functional group into the PAN network. Notably, hydrophobic–hydrophilic balance and nitrile–nitrile interactions are impacted by the presence of –COOH in a poly(acrylonitrile-*co*-acrylic acid) poly(AN-*co*-AA). In turn, pervaporation and antifouling characteristics are produced for wastewater treatment. A key adhesive property stimulated by acrylic acid for modification of poly(AN-*co*-AA) is to replace the specific functional group exclusively.^26^ In order to boost the adsorption capabilities, there must be polymer surface modification (physical and chemical).^28^ Because of its unique structure, amidoxime has received considerable attention as a way to improve the surface properties of adsorbents for larger adsorption capacity. This would produce greater efficiency in acidic, as well as alkaline conditions, due to the fact that it is amphoteric with acidic oxime and basic amino groups. Amidoxime was selected as the modifier across functional groups because of the significant electron donor atom content, as well as the capacity to join the pendant adsorbent chain. As an example, Zhao *et al.* synthesized an amidoxime functionalized polystyrene adsorbent, which was applied for the U(vi) ion uptake from aqueous solutions.^29^ Similarly, Lu *et al.* (2019) synthesized silica-supported poly acryl amidoxime for the sorption of Ga(iii).^30^ Xu *et al.* (2017) also prepared amidoxime functionalized acrylonitrile grafted polyethylene for the recovery of uranium.^31^

In this study, the chemical modification on poly(acrylonitrile (AN)-*co*-acrylic acid (AA)) to produce the amidoxime-modified poly(AN-*co*-AA) polymeric adsorbent was carried out by using hydroxylamine hydrochloric (HH) as a modification reagent. The adsorbent was then utilized to capture PNP from aqueous solution. The present study has not been described in any literature yet. Several adsorption variables were examined, including the adsorbent dosage, temperature, reaction time and PNP initial concentration. The kinetics and equilibrium data were investigated in order to investigate the PNP removal mechanism.

## 2. Experimental

### 2.1 Materials and chemicals

Acrylonitrile (CH_2_CHCN) and acrylic acid (C_3_H_4_O_2_) monomers were obtained from (Acros Organics, New Jersey, USA). Potassium persulphate (KPS) and sodium bisulphate (SBS) (R&M Chemicals, Essex, UK) were used for the free radical polymerization. Acrylonitrile and acrylic acid were purified by passing through aluminum oxide to eliminate impurities. The copolymerization reaction medium used was deionized water. Hydroxylamine hydrochloride (NH_2_OH·HCl) (Acros Organics, New Jersey, USA) was used for chemical modification. Sodium carbonate (Na_2_CO_3_), sodium hydroxide (NaOH) and hydrochloric acid (HCl) were obtained from R&M Chemicals, Essex, UK. Methanol and ethanol were purchased from R&M Chemicals, Essex, UK. The target analyte used was *p*-nitrophenol (C_6_H_5_NO_3_), which was obtained from Acros Organics, New Jersey, USA. The chemicals used were of analytical grade, and applied as received with no additional purification (unless stated).

### 2.2 Poly(acrylonitrile-*co*-acrylic acid) synthesis

The redox polymerization method was applied for the poly(AN-*co*-AA) synthesis, and the reaction medium of 200 mL of deionized water was purged through the injection of N_2_ gas for half an hour. Then, acrylonitrile monomer (95% v) and (5% v) acrylic acid were added to the reaction medium, alongside initiators (sodium bisulphate (2.09 g) and potassium persulphate (2.16 g)), in a 500 mL three-necked round-bottom flask with a water condenser attached. The reaction was stirred continuously at 200 rpm at 60 °C for 2 hours under an N_2_ atmosphere. The polymerization produced slurry that was then precipitated in 100 mL of methanol for 60 min. Following this, the polymer formed was washed with methanol/deionized water until a pH of ∼7 was reached. Next, the polymer was dried in an oven under 45 °C until a constant weight was reached. This is a slightly modified redox polymerization method from the previously employed synthesis route,^26,32^ due to the application of a shorter time and lower temperature.

### 2.3 Amidoxime modification of poly(AN-*co*-AA)

**Fig. 1:**
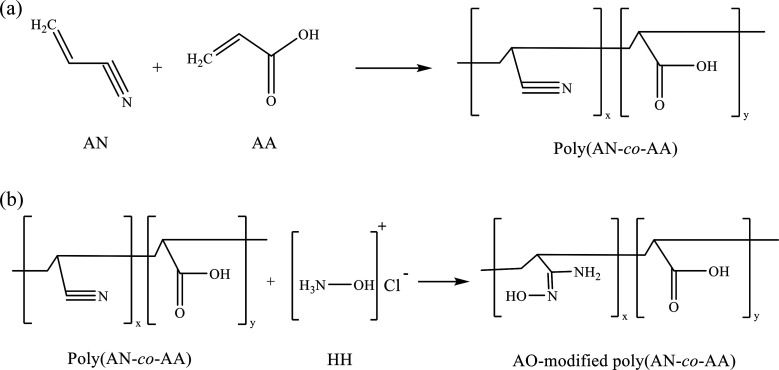
(a) Redox copolymerization of acrylonitrile (AN) and acrylic acid (AA) to produce poly(AN-*co*-AA); (b) modification of poly(AN-*co*-AA) with hydroxylamine hydrochloride to produce the amidoxime (AO)-modified poly(AN-*co*-AA).

The chemical modification was carried out by dissolving 4.00 g of hydroxylamine hydrochloride (NH_2_OH·HCl) into 140 mL 1 : 1 (v/v) ethanol/deionized water in a 250 mL three-necked round-bottom flask under an N_2_ atmosphere. The mixture was stirred over half an hour, and the temperature was gradually increased to 60 °C. Following this, the pH value of the mixture was adjusted to 8 by adding ∼2.95 g sodium carbonate. Then, 2.00 g of poly(AN-*co*-AA) polymer was brought into the solution under reflux. The mixture was stirred at 200 rpm, and the reaction was carried out at 70 °C for 4 h. The output of the amidoxime-modified poly(AN-*co*-AA) was then filtered, and washed with ethanol and deionized water before being dried at 50 °C in an oven for 24 h. Fig. 1 shows the molecular structure of the copolymer, as well as the amidoxime (AO)-modified copolymer.

### 2.4 Experimental procedures for PNP removal

In order to examine the effect adsorption process parameters, kinetics, isotherm and thermodynamics have, batch adsorption experiments were undertaken. To achieve optimal results, the one variable at a time (OVAT) approach was used. The stock solution of PNP was developed through dissolving the necessary mass of PNP particles in distilled water, while a number of experimental concentrations were produced through diluting the necessary stock solution volume. The pH level was maintained at 7 by adjusting the solution with NaOH solution (0.1 M) and HCl solution (0.1 M). The experiments were conducted using 250 mL Erlenmeyer flasks containing 100 mL of PNP solution with the intended concentration and known AO-modified poly(AN-*co*-AA) dose in a temperature-controlled incubator shaker (ST-200R, SASTEC) at 150 rpm. The incubator was properly covered with black fabric during the entire experiments in order to mitigate the possibility for photo-oxidation of PNP. At pre-determined times, the samples were taken out, filtered, and their PNP concentration was evaluated through a 10 mm quartz cell in a UV-Vis spectrometer (Shimadzu UV-1800, Japan) at a wavelength of 317 nm. Eqn (1) and (2) describe the level of PNP uptake (*R*_p_, %) and quantity of PNP adsorbed (*q*_e_) onto AO-modified poly(AN-*co*-AA), respectively:1
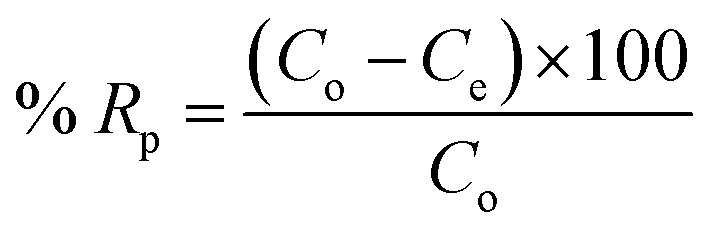
2
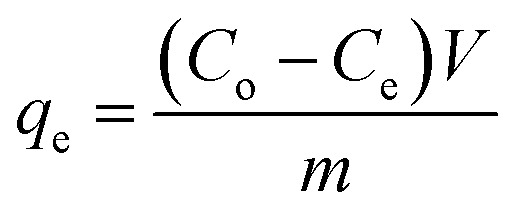
where *C*_o_ and *C*_e_ are the liquid-phase concentrations (mg L^−1^) of PNP at the initial point and at equilibrium, respectively. *V* (L) is the solution volume and *m* (g) is the mass of the adsorbent used.

Desorption studies were conducted, with agitation of 0.2 g of PNP saturated AO-modified poly(AN-*co*-AA) with 20 mL of 95% ethanol for two hours. Then, the regenerated adsorbent was filtrated, washed and oven-dried at 50 °C for 24 hours. Eqn (3) was applied to calculate the regeneration efficiency.3
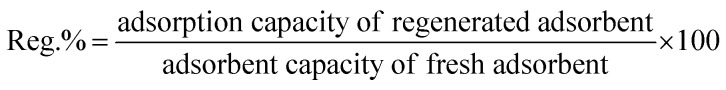


### 2.5 Kinetics and isotherm studies

Pseudo first order,^33^ pseudo second order,^34^ Elovich^35^ and intraparticle diffusion^36^ models were used to study the kinetic behavior during the adsorption. Furthermore, isotherm analysis was conducted through different PNP concentrations from 20 mg L^−1^ to 200 mg L^−1^ at equilibrium time. In turn, the equilibrium data were input into the Langmuir,^37^ Freundlich,^38^ Temkin^39^ and Redlich–Peterson^40^ isotherms, allowing for calculation of the model constants. Table 1 shows the non-linear models that were applied with the Solver Add-ins for Microsoft Excel spreadsheets. This is achieved by defining the objective, in order to limit the sum of squares error (SSE) and altering the kinetic and isotherm parameter variables. Eqn (4) was used to estimate the SSE value:^41^4
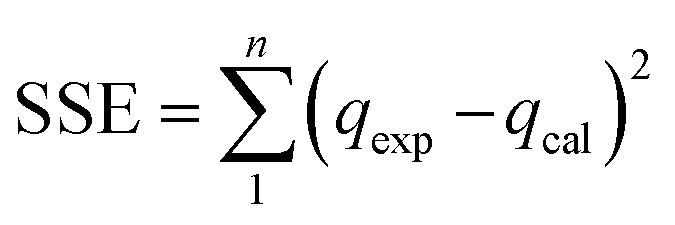


Adsorption kinetics and isotherm models

**Table:** 

Kinetic models	Equations	Parameters
Pseudo-first order	*q*_*t*_ = *q*_e_(1 − e^−*k*_1_*t*^)	*K*_1_: pseudo-first-order rate constant (min^−1^)
Pseudo-second order	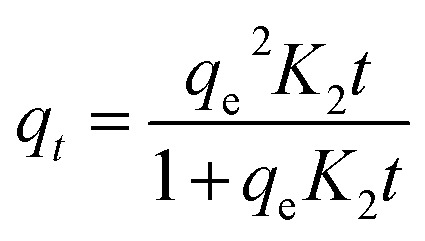	*K*_2_: pseudo-second-order rate constant (g mg^−1^ min^−1^)
Elovich	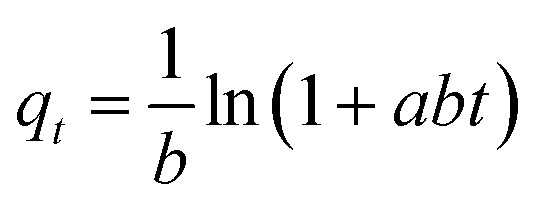	*a*: initial adsorption rate constant (mg g^−1^ min^−1^), *b*: desorption rate constant (g mg^−1^)
Intra-particle diffusion	*q*_*t*_ = *K*_ip_*t*^0.5^ + *C*_ip_	*K*_ip_: intra-particle diffusion rate constant (mg g^−1^ min^−0.5^), *C*_ip_: constant related to boundary layer thickness (mg g^−1^)

**Table:** 

Adsorption isotherms	Equations	Parameters
Langmuir	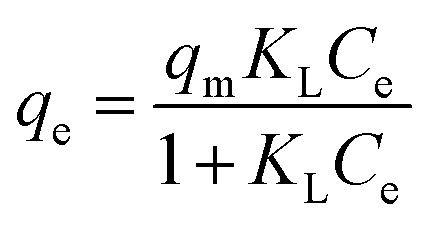	*q*_m_: monolayer capacity (mg g^−1^), *K*_L_: Langmuir constant (L mg^−1^)
Freundlich	*q*_e_ = *K*_F_*C*_e_^1/*n*^	*K*_F_: Freundlich constant (mg g^−1^) (L mg^−1^)^1/*n*^, *n*: adsorption intensity
Temkin	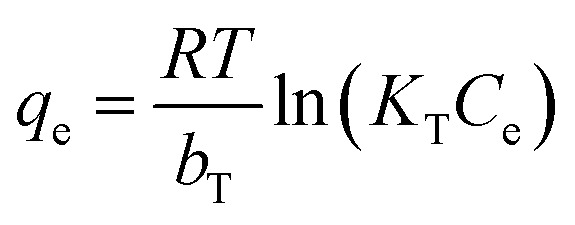	*K*_T_: Temkin constant (L mg^−1^), *b*_T_: adsorption intensity (J mol^−1^)
Redlich–Peterson	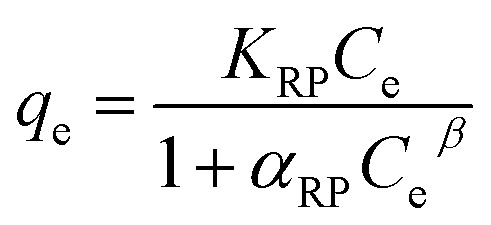	*K*_RP_: R–P constant related to adsorption capacity (L g^−1^), *α*_RP_: binding site affinity (mg^−1^), *β*: isotherm exponent

Furthermore, a dimensionless constant called the equilibrium parameter (*R*_L_) was used to determine the nature of the adsorption process.^42^ The equilibrium parameter *R*_L_ is expressed by the following equation.
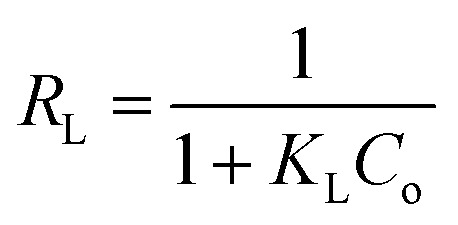
where *C*_o_ and *K*_L_ are the initial concentration of PNP (mg L^−1^) and Langmuir adsorption constant (L mg^−1^), respectively.

### 2.6 Characterization of adsorbents

The characterization of poly(AN-*co*-AA), AO-modified poly(AN-*co*-AA) and PNP-loaded AO-modified poly(AN-*co*-AA) was carried out by using the Fourier transform infrared spectrometer (FTIR) (1750X-PerkinElmer, Inc., Waltham, MA, USA), ranging from 4000–650 cm^−1^. The X-ray diffractometer (XRD) was used to evaluate the samples' crystal structures (Shimadzu, model XRD-D6000). The scanning electron microscopy (SEM) analysis of the particles was carried out by using a Hitachi S-3400N instrument, with an acceleration voltage of 10–20 kV and a magnification of 3000× and resolution of 10 μm (Hitachi S-3400N High-Technologies Corporation, Minato, Tokyo, Japan). Then, the elemental composition of the samples was estimated through a LECO CHN-932 (Leco Corporation, St. Joseph, MI, USA).

## 3. Results and discussion

### 3.1 Characterization of AO-modified poly(AN-*co*-AA)

**Fig. 2:**
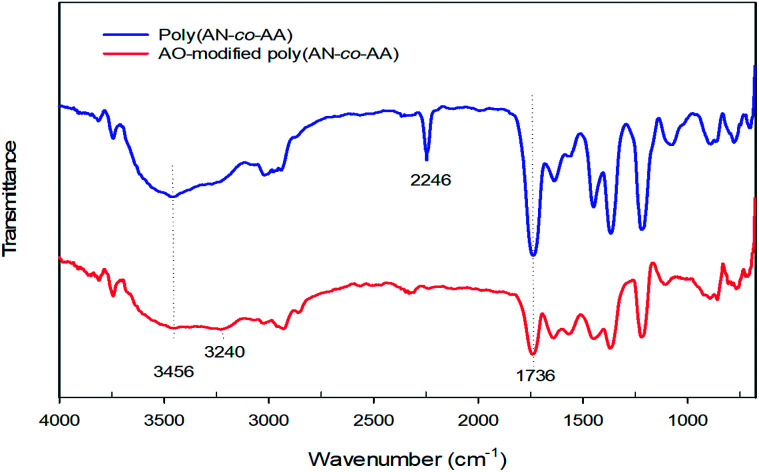
FTIR spectra of poly(AN-*co*-AA) (blue) and AO-modified poly(AN-*co*-AA) (red).

Fig. 2 shows the Fourier transform infrared (FTIR) spectra of poly(AN-*co*-AA), and AO-modified poly(AN-*co*-AA). Notably, the 1736–1739 cm^−1^ bands for both samples describe the C=O stretching vibration, which denotes that there was successful co-polymerization for AA and AN.^43,44^ In the spectra of AO-modified poly(AN-*co*-AA), we observed a decrease in intensity of the C=O stretching vibrations due to the presence of a functional group (AO) on the polymer surface. For the non-modified polymer, there was an appearance of an absorption band at 2246 cm^−1^ due to the stretching vibration of the nitrile group from the acrylonitrile unit in the copolymer chain, which would later disappear following modification.^45^ In addition, peaks at 2927–2938 cm^−1^ are shown in both spectra, which is due to the stretching of the asymmetric C–H band.^46^ Furthermore, the O–H designated peak at 3456 cm^−1^ and the presence of the 3240 cm^−1^ band were noted in the spectra, which are typical frequencies for the O–H and N–H_2_ functional groups, respectively.^47,48^ This is due to the development of amidoxime groups from the nitrile moieties along the copolymer chains.^49^ In addition, the absorption bands in the region of 1640–1564 cm^−1^ appearing in AO-modified poly(AN-*co*-AA) might be attributed to the stretching vibration of the C=N groups.^47^ Earlier work has presented similar results regarding the characteristic bands for the amidoxime functionalized adsorbents.^50–52^

**Fig. 3:**
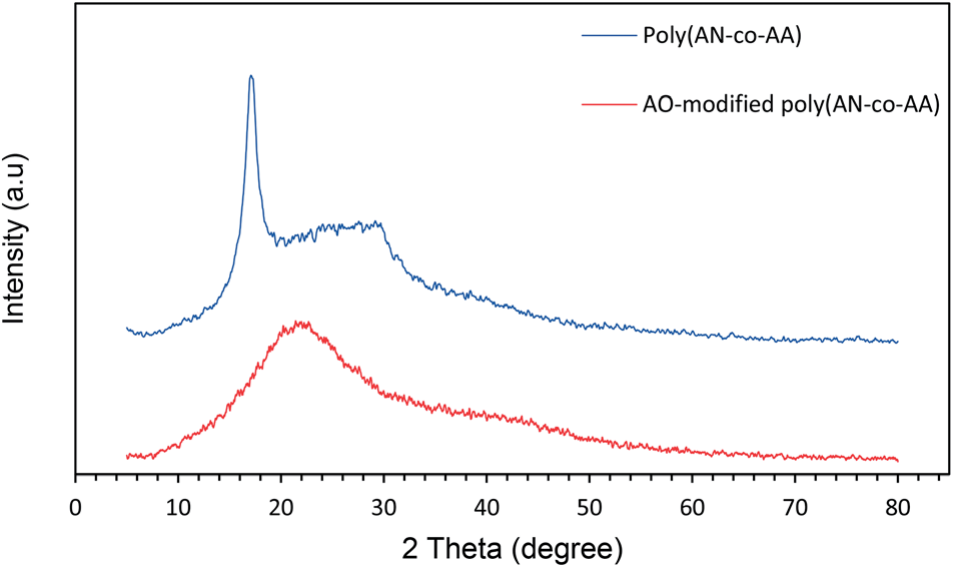
XRD pattern of poly(AN-*co*-AA) and AO-modified poly(AN-*co*-AA).

The XRD patterns of poly(AN-*co*-AA) and AO-modified poly(AN-*co*-AA) are displayed in Fig. 3. The intercalation was carried out by comparing the interlayer spacing *d*(001) of poly(AN-*co*-AA) and AO-modified poly(AN-*co*-AA) by using the Bragg formula. The equation used is as follows:

5 2*d* sin *θ* = *nλ*

where *d* is the average interlayer spacing, *θ* is the half diffraction angle, *n i*s the diffraction series, and *λ* is the wavelength of the X-ray beam. The 2*θ* value is smaller when the interlayer spacing is greater. As the 2*θ* value of 16.92° is used, the interlayer spacing of *d*(001) in poly(AN-*co*-AA) is calculated as 5.23 Å by substitution in eqn (5). On the other hand, there was no crystal order shown in the XRD patterns of AO-modified poly(AN-*co*-AA). The wider peaks of 2*θ* = 13–30° revealed their amorphous nature.^53^ Similar results were observed by Sulu *et al.* (2017) in the study of a series of nanocomposite stereoregular hybrid gels based on graphene oxide and poly(*N*-isopropylacrylamide).^90^ Thus, this study indicates the even dispersion of poly(AN-*co*-AA) into the main body of AO-modified poly(AN-*co*-AA).

**Fig. 4:**
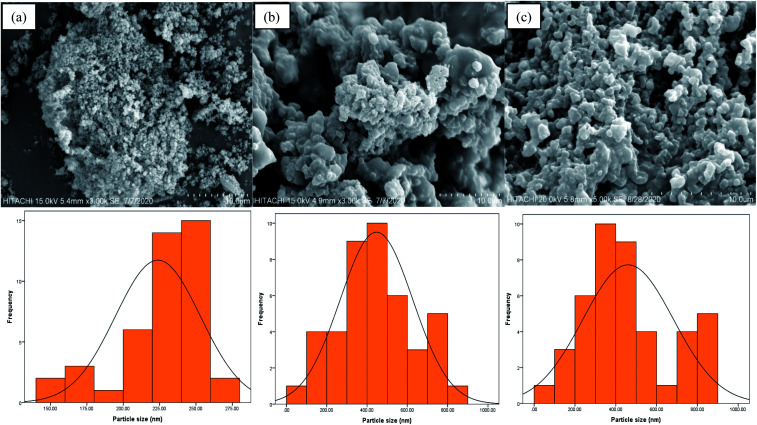
SEM micrographs of (a) poly(AN-*co*-AA), (b) AO-modified poly(AN-*co*-AA), and (c) PNP loaded AO-modified poly(AN-*co*-AA).

Fig. 4(a–c) describe the respective surface morphologies of poly(AN-*co*-AA), AO-modified poly(AN-*co*-AA), and PNP-loaded AO-modified poly(AN-*co*-AA). Fig. 4(a and b) show the spherical particles together with an agglomerated morphology and coarse surface. The agglomerated/clustering of the polymeric particles could be related to the complex formation due to the polymerization mechanism and solution viscosity. The microsphere diameters and particle size distributions were calculated using ImageJ software from the SEM image analysis of 100 individual particles.^54^ For poly(AN-*co*-AA), the average particle size is 225–260 nm, whereas AO-modified poly(AN-*co*-AA) has an average particle size of 300–500 nm. This notable difference denotes that the amidoxime group was incorporated into the synthesized copolymer.^26,55^ In addition, the mass transfer of PNP molecules to the AO-modified poly(AN-*co*-AA) surface would be boosted by the coarse surface and accessible pores through the provision of binding spots. After PNP adsorption (see Fig. 4(c)), the PNP loaded AO-modified poly(AN-*co*-AA) surface was packed, with the pores becoming less available, as stimulated by PNP coverage and the connection with the particle surfaces.

Table 2 displays the elemental composition and textural characteristics of the poly(AN-*co*-AA) and AO-modified poly(AN-*co*-AA) polymer samples. Through elemental analysis, there was a lower carbon count found in the modified polymer compared to the unmodified polymer, as well as higher hydrogen and nitrogen content. This is evidence of the nitrile group substitution with N–H_2_ attachment in the C≡N group through the significant rise in nitrogen composition from 23.91% (poly(AN-*co*-AA)) to 27.47% (AO-poly(AN-*co*-AA)). The substantial change seen supports the notion of the amidoxime moiety and effective functionalization in the nitrile group. The work of ref. 56 present analogous findings, supporting the presence of an efficient modification process of nano-magnetic cellulose composite. Subri and co-workers reported a similar observation, in which there was an increment of nitrogen content after chemical modification of the hypercrosslinked poly(acrylonitrile-*co*-divinylbenzyl-80-*co*-vinylbenzyl chloride) terpolymer with ethylenediamine (EDA) due to the presence of the secondary amino group in EDA.^54^

**Table:** Elemental composition analysis of poly(AN-*co*-AA) and AO-modified poly(AN-*co*-AA)

Element(s)	Elemental composition (%)	
	Poly(AN-*co*-AA)	AO-modified poly(AN-*co*-AA)
Carbon (wt%)	63.95	36.12
Hydrogen (wt%)	5.870	6.828
Nitrogen (wt%)	23.91	27.47

### 3.2 Effects of the adsorption parameters

#### 3.2.1 Effect of the polymer dosage

**Fig. 5:**
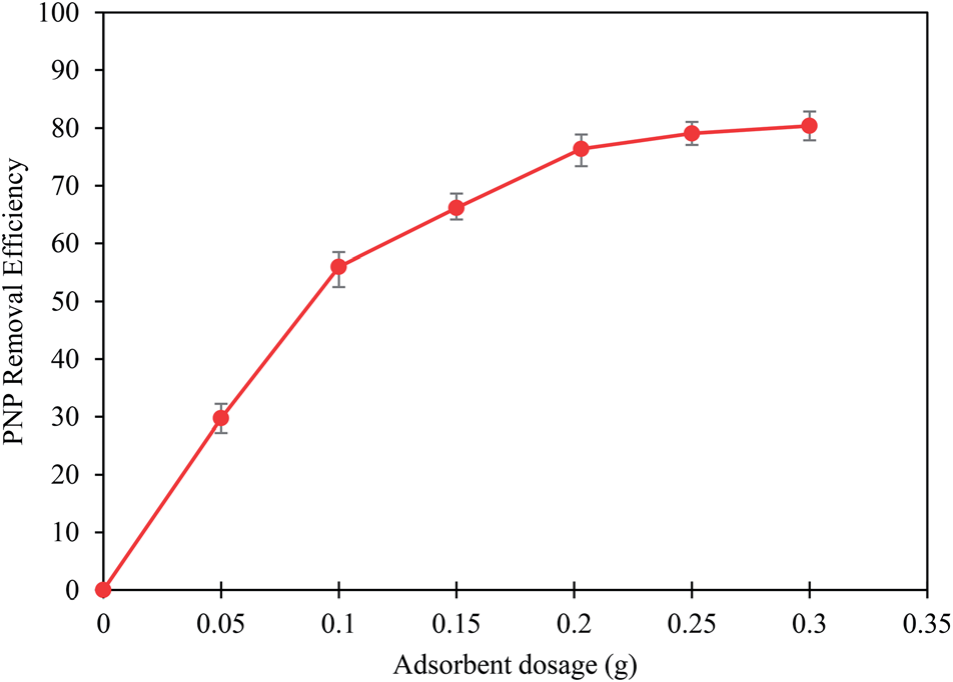
Effect of the adsorbent dosage on the PNP removal efficiency (*C*_o_: 50 mg L^−1^; time: 60 min; temperature: 298 K; 150 rpm).

Crucially, the amount of adsorbent affects the cost and efficiency of prediction in the adsorption process. The impact of AO-modified poly(AN-*co*-AA) dosage on PNP removal levels is displayed in Fig. 5. It showed that the PNP removal percentage increased from 29.7% to 80.4% as the adsorbent dosage was increased from 0.05 g to 0.30 g. This is due to the fact that there was a greater number of empty sites for solute entrapment as the adsorbent dosage increased.^57,58^ In addition, the highest removal was achieved at 76.4% with the adsorbent mass of 0.20 g. The increment of adsorbent dosage resulted in minor effects to the PNP removal. This is due to the overlapping of the adsorbent as a result of suspended excess adsorbent particles and hence fewer available active sites.^59^ Therefore, the AO-modified poly(AN-*co*-AA) dose of 0.2 g was confirmed to be the optimal quantity with which further tests would be undertaken.

#### 3.2.2 Effect of temperature

**Fig. 6:**
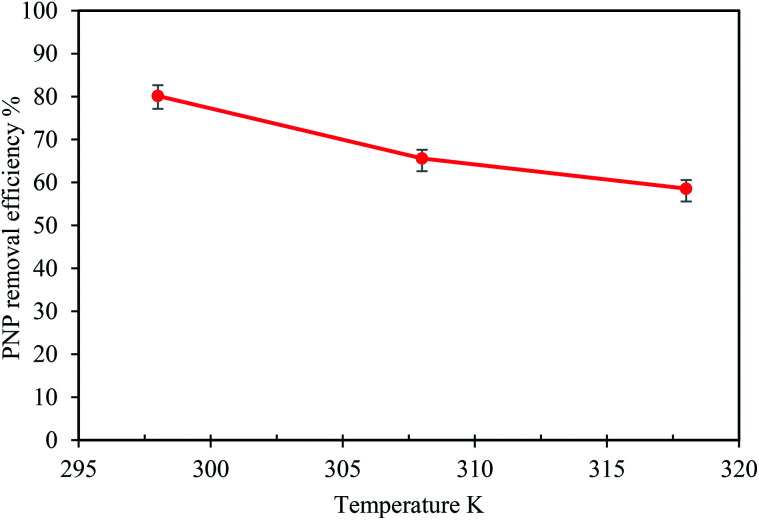
Effect of temperature on the adsorption of PNP onto AO-modified poly(AN-*co*-AA) (adsorbent dose: 0.2 g/100 mL; *C*_o_: 50 mg L^−1^; pH: 7; time: 60 min).

Temperature may greatly influence the affinity between the adsorbent and PNP molecules. Therefore, the impact of the solution temperature on the level of PNP removal as a result of the AO-modified poly(AN-*co*-AA) adsorbent was examined at temperatures of 298 to 318 K. Fig. 6 shows the results of the temperature impacts, where a lower temperature is clearly preferable for greatest PNP adsorption onto AO-modified poly(AN-*co*-AA) (80.15%), while PNP sequestration would be lower when the solution temperature increased up to 318 K. This highlights the exothermic properties of the adsorption process. In most cases for exothermic adsorption functions, raising the temperature brings about desorption of the target pollutant to the fluid phase, at equilibrium. In turn, the temperature increased, the diffusive mass transfer and solubility of PNP in water become greater, producing weakness for the adsorptive forces amongst adsorbent sites. This would result in less physical adsorption and worse removal efficiency.^18,60^ These findings were, once again, in line with the work of Yang *et al.*, which described the adsorptive removal of PNP through a designed, hierarchical porous carbon from semi-coke *via* a facile preparation method for *p*-nitrophenol adsorption. Due to the finding in the present work, further adsorption analysis in this study was carried out at 298 K.

#### 3.2.3 Effect of the initial concentration and contact time

**Fig. 7:**
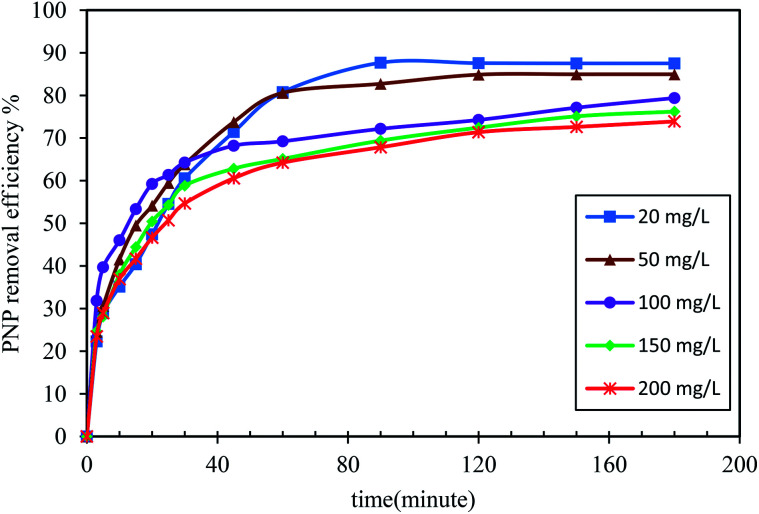
Effect of the residence time at various concentrations (20–200) mg L^−1^ to PNP adsorption by AO-modified poly(AN-*co*-AA) (adsorbent load: 0.2 g/100 mL; initial pH: 7; temperature: 298 K; agitation speed: 150 rpm).

As shown in Fig. 7, the effect of the contact time on adsorption was examined with different initial PNP concentrations (between 20 and 200 mg L^−1^). The other parameters were set at 0.20 g of the adsorbent mass at 298 K, and solution pH at 7.0. The total PNP adsorption by AO-modified poly(AN-*co*-AA) was greater when the contact time was increased, and then remained constant once the contact time was equal to the equilibrium time. At first, the uptake quickly became greater across the first five minutes, denoting that many active sites were available. As time passed, the solute uptake was slower, and this might be due to less available adsorption sites. Conversely, as the concentration of the solute became greater, the adsorption also increased, as explained by the significant PNP concentration at the start, providing the required driving force to surpass the mass transfer resistances across the PNP solution and AO-modified poly(AN-*co*-AA) surface (which is the resistance from the water phase to the adsorbent surface). Previously, Jawad and co-workers showed similar results on dye removal by carbon modified chitosan,^61^ and a similar observation was reported for the initial concentration of the adsorption of phenol by activated carbon.^62^ In most cases, when the initial concentrations became greater, then the adsorption capacity also increased up to a point of critical initial concentration, where the adsorbate is rapidly saturated into the surface and the adsorbent interlayer.

### 3.3 Adsorption kinetics studies

**Fig. 8:**
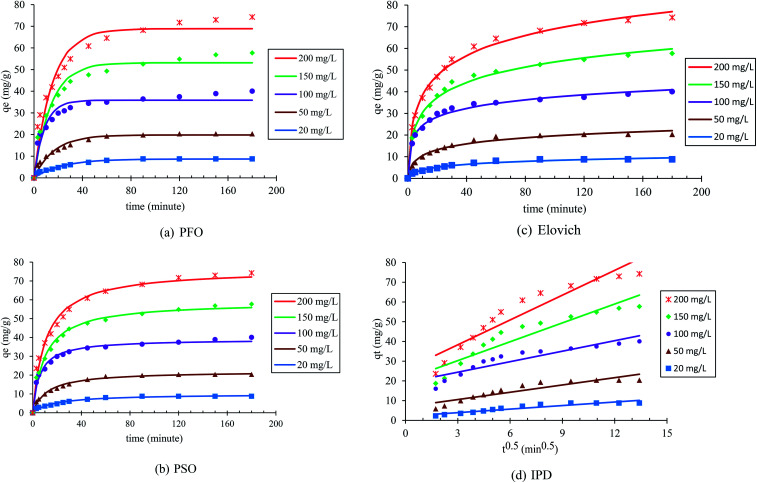
Pseudo-first order (a), pseudo-second order (b), Elovich (c) and intra-particle diffusion (d) kinetic models for the adsorption of PNP on AO-modified poly(AN-*co*-AA) at various concentrations.

The adsorption kinetics was investigated to offer adequate data regarding the adsorption process, and to describe the adsorption reaction type of *p*-nitrophenol. Table 1 describes the kinetic models used to investigate the experimental findings of various initial PNP concentrations. By examining the kinetic profiles, data regarding the time necessary to achieve equilibrium were produced, as well as information related to the material stability, and how it interacts with the targeted solute and on the controlling step in the sorption process. Fig. 8 and Table 3 display the fitting outcomes for the four kinetic models in question. In most cases, the contrasting values of the correlation coefficients *R*^2^ describe the correlation of the experimental data and the model-predicted values. It is clear that all models (previously mentioned) are adequately fitted with the highest correlation coefficient value *R*^2^. The kinetics data were found to be perfectly fitted with the pseudo-second order (PSO) (*R*^2^ ≥ 0.980) and Elovich (*R*^2^ ≥ 0.975), as summarized in Table 3. The agreement with the PSO model indicates that the adsorption rate of PNP onto AO-modified poly(AN-*co*-AA) was influenced by surface-active sites existing in the adsorbents, and the adsorption process was controlled by chemisorption.^63^ In addition, the way that the adsorption of PNP on AO-modified poly(AN-*co*-AA) occurs can be seen through the Elovich model, which denotes that adsorption is a diffusion process of a complicated heterogeneous stage, where the adsorbent surface energy distribution was uneven and chemical adsorption occurred.^64^

**Table:** The parameters of the kinetics models for PNP adsorption on AO-modified poly(AN-*co*-AA)

PNP initial concentration (mg L^−1^)	20	50	100	150	200
*q*_exp_ (mg g^−1^)	8.771	20.380	40.050	57.690	74.260

**Pseudo-first order**					
*q*_cal_ (mg g^−1^)	8.705	19.810	35.859	53.139	68.873
*K*_1_ (min^−1^)	0.044	0.060	0.117	0.071	0.066
*R* ^2^	0.973	0.972	0.940	0.959	0.951
SSE	3.456	17.829	109.576	183.660	374.924

**Pseudo-second order**					
*q*_cal_ (mg g^−1^)	9.962	22.063	39.079	58.931	76.323
*K*_2_ (g mg^−1^ min^−1^)	0.006	0.004	0.005	0.002	0.001
*R* ^2^	0.980	0.990	0.986	0.989	0.984
SSE	2.323	5.556	22.825	42.784	104.114

**Elovich**					
*a* (mg g^−1^ min^−1^)	1.151	4.945	39.763	18.193	21.517
*b* (g mg^−1^)	0.487	0.245	0.174	0.096	0.073
*R* ^2^	0.975	0.981	0.989	0.991	0.995
SSE	2.685	9.948	16.995	33.594	33.468

**Intraparticle diffusion model** a					
*C*_ip_ (mg g^−1^)	2.112	6.850	19.094	20.720	25.725
*K*_ip_ (mg g^−1^ min^−0.5^)	0.598	1.232	1.773	3.186	4.192
*R* ^2^	0.884	0.846	0.837	0.872	0.895

a Represented the calculated parameters derived from the linear model.

Moreover, a crucial stage is to establish the adsorption process mechanism, in order to allow the process to be designed and controlled optimally. Overall, adsorption occurs in four key phases. First, the adsorbate moves from the solution to the layer next to the adsorbent particles. During the following two phases, the molecules pass through the layer to the adsorbent particles, and through the adsorbent surface pores. Finally, the adsorbent fully adsorbs the adsorbate. Notably, the rate-limiting step is a stage, or multiple stages, which manage the adsorption process. Stages 1 and 4 are often rapid, and are not considered when trying to establish the rate-limiting step.^4,65^ In order to find whether the rate-limiting step is the second or third stage, the diffusion rate of PNP onto AO-modified poly(AN-*co*-AA) was examined using the intraparticle diffusion model. The rate-limiting step was applied when the plots are linear and pass through the origin, moving the adsorbate molecules from the solution to adsorbent. In the case where the plot is non-linear and the origin is not passed through, then the rate-limiting step includes additional stages, including pore diffusion, intra-particle diffusion and chemical reaction in the surface.^66^

Fig. 8 presents the linearized plots of the Weber and Morris model, which failed to pass through the origin, with a *R*^2^ value of (<0.9). This denotes that the intraparticle diffusion mechanism was not the single rate-limiting step for the adsorption of PNP by AO-modified poly(AN-*co*-AA). Thus, it might be possible that the adsorption was impacted by a greater number of processes. The adsorption involved surface adsorption, as well as intraparticle diffusion, during the PNP adsorption by the AO-modified poly(AN-*co*-AA) polymer. These findings are similar to those in ref. 67, which examined phenol adsorption by using commercial activated carbon.

### 3.4 Adsorption isotherm studies

**Fig. 9:**
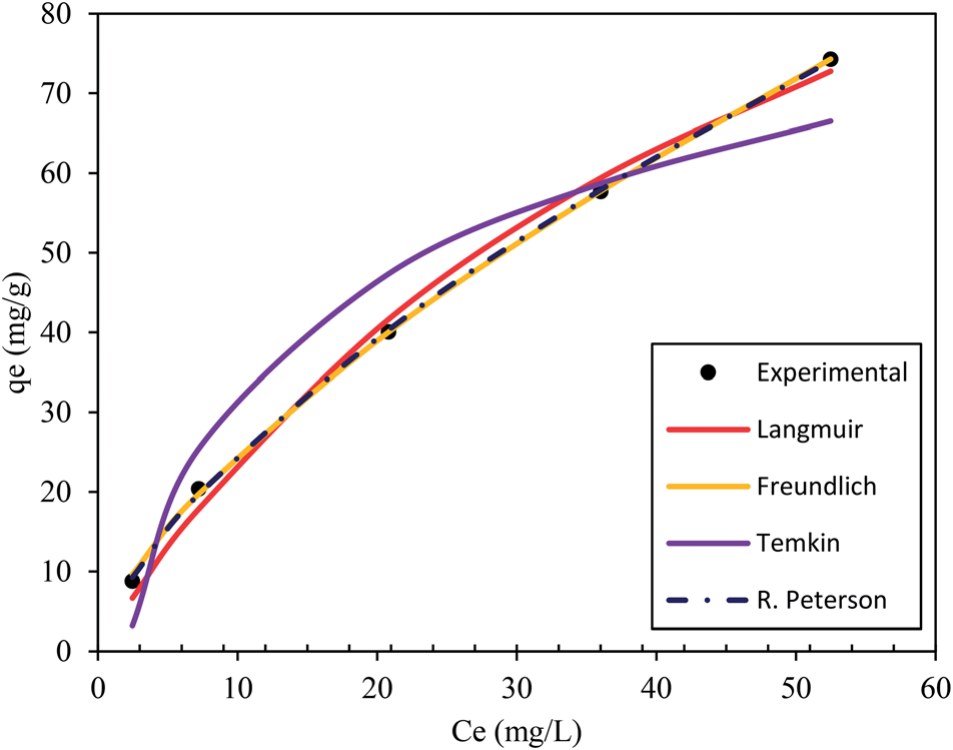
Isotherm model plots of the Langmuir, Freundlich, Temkin, and Redlich–Peterson models for the adsorption of *p*-nitrophenol on AO-modified poly(AN-*co*-AA) at 298 K (pH 7, adsorbent dose = 0.2 g, volume of solution = 100 mL, and agitation speed = 150 rpm).

Isotherm studies provide the relationship on the adsorption equilibrium of the adsorbate onto the adsorbent surface. It is used to define the distribution of adsorption molecules between the liquid phase and solid phase once an equilibrium is reached in the adsorption process. A suitable level of comprehension of adsorption isotherms is necessary in order to make positive adjustments to the adsorption mechanism pathways and for the adsorption system to be designed optimally.^68^ The non-linear model of the Langmuir, Freundlich, Temkin and Redlich–Peterson models that are applied in this study are shown in Fig. 9. Table 4 shows the related isotherm parameters, presenting a robust correlation coefficient *R*^2^ (0.940–0.999) across all models. Based on the greatest correlation coefficient value *R*^2^ and lowest SSE values, it was shown that PNP adsorption had the best fit with the Redlich–Peterson model, with the *R*^2^ of (0.999) and SSE of (0.880). These values describe a sorption process involving Langmuir, as well as Freundlich isotherms. Thus, it can be put forward that the PNP uptake onto AO-modified poly(AN-*co*-AA) is made up of the monolayer and complexation interactions for the current context. The exponent value of the Redlich–Peterson isotherm model (*β*) was shown to be closer to zero, based on the Freundlich isotherm model with its substantial *R*^2^ (0.999).^69^ Additional evaluations were conducted in order to determine how appropriate the monolayer adsorption and surface homogeneity were through estimating the separation factor, *R*_L_. The value of 0 < *R*_L_ < 1 indicates favorable adsorption, while *R*_L_ > 1 denotes unfavorable adsorption. When *R*_L_ = 1, then a linear adsorption is in place, while *R*_L_ = 0 describes irreversible adsorption. Table 4 shows that the values of *R*_L_ (0.200–0.714) were between zero and 1, which demonstrates favorable sorption.^70^ In addition, 1/*n* describes the adsorption intensity under the Freundlich model, and specifies the type of adsorption as favorable (0 < 1/*n* < 1), unfavorable (1/*n* > 1) and irreversible (1/*n* = 1).^71^ For the current context, 1/*n* is between 0 and 1, which is favorable. This is a way of estimating the adsorption intensity or surface heterogeneity, which increases as the value moves towards zero. According to the calculation using the Temkin model, the heat was dissipated at (119.56 J mol^−1^) through the adsorption process. This indicates that the adsorption is an exothermic process. Such observation was also reported on the functionalized multiwalled carbon nanotubes for the removal of the dye.^72^

The parameters of the isotherm models and equilibrium parameter (*R*_L_) for PNP adsorption on AO-modified poly(AN-*co*-AA)

**Table:** 

Isotherm model(s)	Parameters		*R* ^2^
Langmuir	*q*_max_ (mg g^−1^)	143.06	0.994
	*K*_L_ (L mg^−1^)	0.020	
	SSE	18.785	
Freundlich	*K*_F_ (mg g^−1^) (L mg^−1^)^1/*n*^	5.257	0.999
	*n*	1.495	
	SSE	1.172	
Temkin	*K*_T_ (L mg^−1^)	0.472	0.940
	*b*_T_ (J mol^−1^)	119.557	
	SSE	170.959	
Redlich, D. L. Peterson	*K*_RP_ (L g^−1^)	13.444	0.999
	*α* (mg^−1^)	1.821	
	*β*	0.389	
	SSE	0.880

**Table:** 

	*C*_o_ (mg L^−1^)	*R*_L_
Separation factor (*R*_L_)	20	0.714
	50	0.500
	100	0.333
	150	0.250
	200	0.200

### 3.5 Thermodynamic studies

Thermodynamic characteristics were established to investigate the spontaneity of the adsorption process. The change in enthalpy (Δ*H*°), free energy (Δ*G*°) and entropy (Δ*S*°) were examined for the determination of the thermodynamic properties for PNP adsorption onto the AO-modified poly(AN-*co*-AA) sorbent. Eqn (6) and (7) were used to calculate the parameters:

6 Δ*G*° = −*RT* ln *K*_d_

**Fig. 10:**
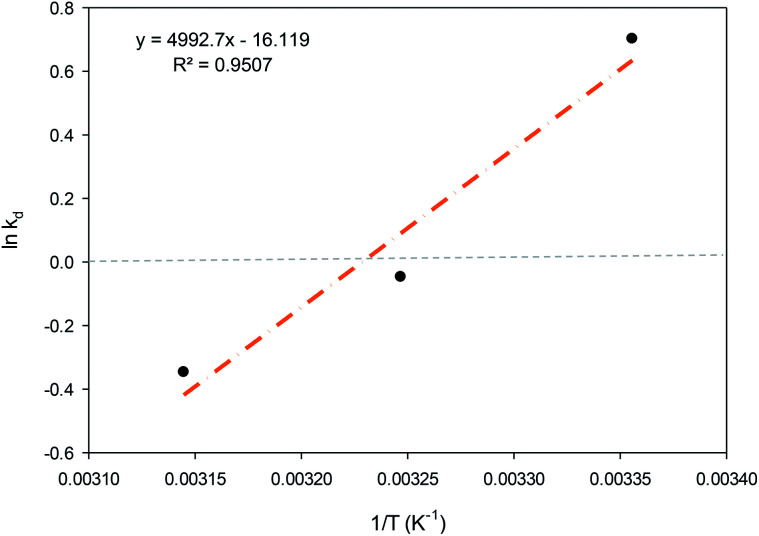
van't Hoff plot for PNP adsorption onto AO-modified poly(AN-*co*-AA) (adsorbent dose = 0.2 g, volume of solution = 100 mL, pH 7, and agitation speed = 150 rpm).

7
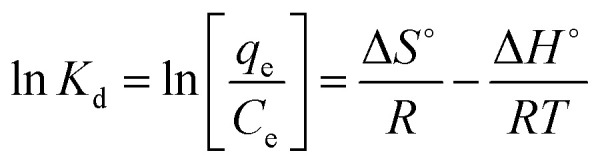
where *T* and *R* are the temperature (K) and the ideal gas constant [8.314 J (mol^−1^ K^−1^)], respectively, and *K*_d_ is the linear sorption distribution coefficient (*q*_e_/*C*_e_). The van't Hoff equation was used to plot the ln *K*_d_*vs.* 1/*T*, (Fig. 10). The Δ*S*° and Δ*H*° values were found through the intercept and slope, respectively. Eqn (7) was used to estimate the free energy (Δ*G*°). Table 5 shows the values of Δ*G*°, Δ*H*° and Δ*S*°.

**Table:** Thermodynamic parameters for PNP adsorption on AO-modified poly(AN-*co*-AA)

*T* (K)	*K*_d_	Δ*G*° (kJ mol^−1^)	Δ*H*° (kJ mol^−1^)	Δ*S*° (kJ mol^−1^ K^−1^)
298	2.018	−1.740 (±0.4)	−41.509	−0.134
308	0.954	0.121 (±0.01)		
318	0.707	0.918 (±0.12)		

The spontaneity of the chemical reaction was found through the Gibb free energy change Δ*G*°. In the current context, the Δ*G*° value was shown to have shifted from −ve to +ve when the temperature of the PNP adsorption process was increased from 298 K to 308 K, denoting that the instance of adsorption is spontaneous, while the desorption rate was greater than the adsorption rate when the temperature was increased. A similar observation was reported before.^45^ As Δ*S*° is negative, it is suggested that the solid–solution interface organization of PNP through adsorption by AO-modified poly(AN-*co*-AA) is less random once the solution temperature is increased. Furthermore, the enthalpy change Δ*H*° was −41.509 kJ mol^−1^ for the sorption process. Since this is negative, it provides additional evidence that the sorption process is exothermic. The work of Elhleli and co-workers presented analogous findings, clarifying the presence of an exothermic adsorption process on *p*-nitrophenol retention by activated carbon.^73^

### 3.6 Regeneration studies

**Fig. 11:**
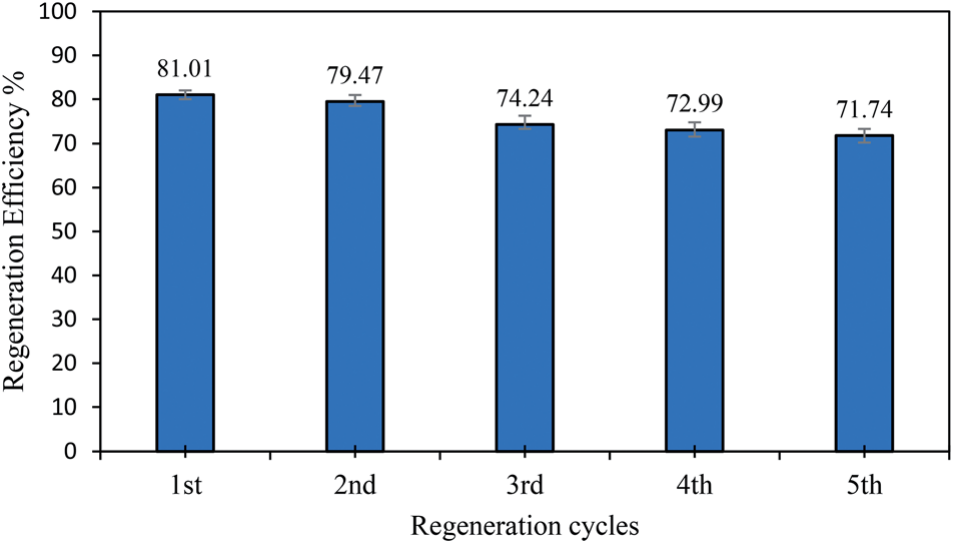
The regeneration study of AO-modified poly(AN-*co*-AA) by using EtOH (adsorbent dosage = 0.2 g/100 mL, *C*_o_ = 50 mg L^−1^, 180 min).

The studies on regeneration into the possibility of the desorbing of PNP molecules from AO-modified poly(AN-*co*-AA) are vital from a financial and environmental standpoint, as well as industrial applicability. In order to achieve AO-modified poly(AN-*co*-AA) regeneration, a green and efficient eluent is considered ideal. The regeneration process required a solvent, and so ethanol was chosen since it is environmentally friendly.^18^ The level of PNP uptake surpassed >71% following five cycles, as depicted in Fig. 11. The desorption of PNP from the surface of AO-modified poly(AN-*co*-AA) was carried out by using ethanol due to the good solubility of PNP in ethanol. The regeneration finding multiple uses of this adsorbent to capture PNP from the aqueous phase is possible by using a common organic solvent (ethanol).

### 3.7 Adsorption mechanism

**Fig. 12:**
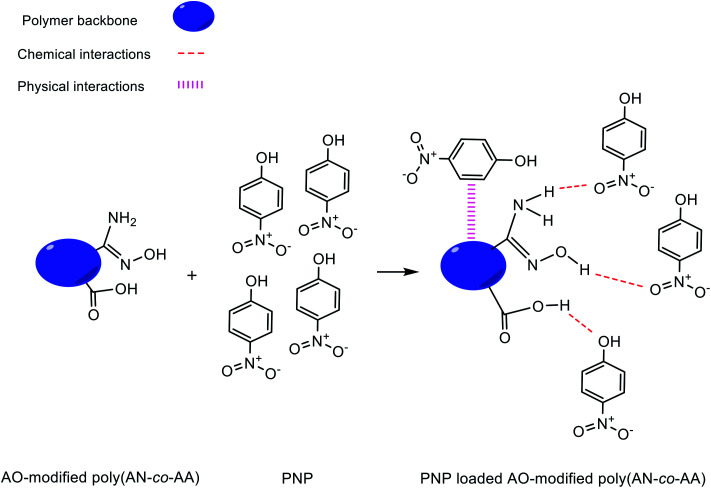
Proposed mechanism of interaction between AO-modified poly(AN-*co*-AA) and PNP.

**Fig. 13:**
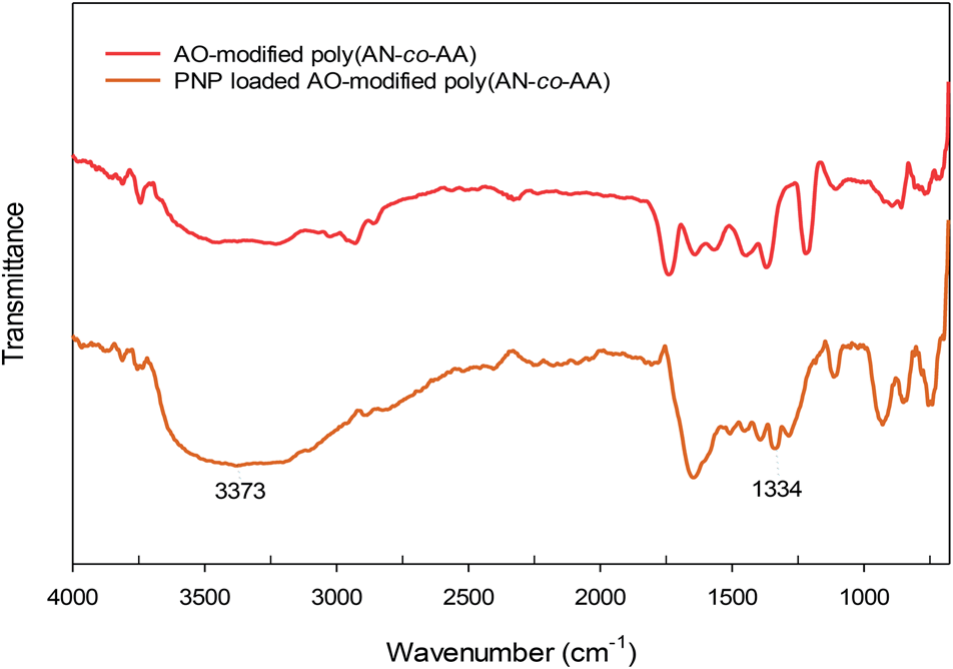
FTIR spectra of AO-modified poly(AN-*co*-AA) and PNP loaded AO-modified poly(AN-*co*-AA).

Fig. 12 describes the proposed adsorption mechanism of PNP onto the AO-modified poly(AN-*co*-AA) surface and from adsorption studies (explained above). At neutral pH condition, PNP in the solution occurred primarily in a molecular form and the adsorption achieved can be explained through physical and chemical bonding interactions. Amidoxime functional groups (–NH_2_, –OH) on the adsorbent surface provide sites where PNP adsorption can occur, with hydrogen bonding between the NH_2_, hydroxyl groups on AO-modified poly(AN-*co*-AA) and the nitro, hydroxyl groups of PNP, as well as physical interactions by van der Waals forces. In addition, the fact that there were functional groups on AO-modified poly(AN-*co*-AA) and significant peaks being altered following PNP adsorption was verified from the FTIR spectra of AO-modified poly(AN-*co*-AA) and PNP loaded AO-modified poly(AN-*co*-AA). As depicted in Fig. 13, the FTIR spectra can be seen with the symmetrical stretching vibration band of aromatic –NO_2_ appearing at 1330 cm^−1^. The peak of 3373 cm^−1^ for the FTIR spectra of PNP was connected, and is assigned to the O–H stretching vibration peak of the phenolic hydroxyl group.^74,75^ Furthermore, the peaks at 1218 cm^−1^ shifted to 1330 cm^−1^ of the PNP loaded AO-modified poly(AN-*co*-AA). A possible reason for this could be the hydrogen bonding among AO-modified poly(AN-*co*-AA) and PNP.^76^ These observations showed that the PNP molecules were successfully adsorbed onto AO-modified poly(AN-*co*-AA) *via* hydrogen bonding between the N–H_2_, hydroxyl groups or carboxyl groups of AO-modified poly(AN-*co*-AA), and the hydroxyl, nitro groups of PNP.

### 3.8 Comparison AO-modified poly(AN-*co*-AA) with other adsorbents

Table 6 displays AO-modified poly(AN-*co*-AA) compared with various other adsorbents, highlighting the fact that the AO-modified poly(AN-*co*-AA) adsorption potential (143.06 mg g^−1^) was noticeably greater than that of the other adsorbents. The AO-modified poly(AN-*co*-AA) that was synthesized in the current study through redox polymerization provides a number of benefits over other approaches. In this study, the polymerization occurred in moderate conditions, which limits the side effects and was completed in a shorter time. Second, the adsorbent has a high regeneration ability up to 71.74% of the regeneration efficiency after 5 cycles of adsorption. As for a comparison with other reported adsorbents in Table 6, the nano zeolite adsorbent has a regeneration efficiency of 70% after 5 cycles of adsorption.^77^ Meanwhile, montmorillonite clay was reported to have a regeneration efficiency of 85% after 4 cycles.^78^ The reported regeneration ability of other adsorbents showed that the AO-modified poly(AN-*co*-AA) adsorbent has a comparable regeneration efficiency with other adsorbents.

**Table:** Comparison of the adsorption capacity of PNP by various adsorbents

Adsorbents	*q*_max_ (mg g^−1^)	References
Microporous AC	185	79
Brazilian peat	23.39	80
Nano zeolite	156.7	77
β-Cyclodextrin nanoporous carbons	100	81
Quaternized β-cyclodextrin–montmorillonite composite	24.56	82
Organo-vermiculite	106.5	83
*Vitis vinifera*	103.1	84
Montmorillonite clay	122.1	78
Ethylenediamine rosin-based resin	82	85
Cauliflower activated carbon-500	37.73	86
Silver(i) 3,5-diphenyltriazolate	184.8	12
Graphene oxide–β-cyclodextrin	117.28	87
**AO-poly(AN-*co*-AA)**	**143.06**	**This study**

## 4. Conclusion

This study described the preparation of amidoxime-modified poly(AN-*co*-AA) adsorbent (in particle form) for the removal of *p*-nitrophenol from an aqueous solution. Furthermore, modified-amidoxime poly(AN-*co*-AA) was employed as the adsorbent to adsorb PNP from the aqueous solution. The optimum adsorption parameters were determined for the PNP molecule adsorption being affected by the AO-modified poly(AN-*co*-AA) dosage, temperature, initial PNP concentration and residence time at 0.20 g/100 mL, 298 K and time = 180 min. Kinetic analyses showed that the factors behind the PNP uptake were mainly described by pseudo-second order, indicating that the adsorption process is in the chemical nature. The isotherm analyses showed that AO-poly(AN-*co*-AA) has the highest sorption capacity with (143.06 mg g^−1^) at 298 K, and was best fitted with both Langmuir and Freundlich models. Furthermore, the exponent value of the Redlich–Peterson isotherm model (*β*) was shown to be closer to zero, based on the Freundlich isotherm model with its substantial *R*^2^ (0.999). The thermodynamic study described the exothermic and spontaneous characteristics of the PNP uptake by AO-modified poly(AN-*co*-AA). Notably, AO-modified poly(AN-*co*-AA) can be regenerated using the environmentally friendly eluent (ethanol) and the extent of PNP adsorption exceeds 71% after five cycles of reusability. The result of this study revealed that AO-modified poly(AN-*co*-AA) can be utilized as a potential adsorbent to remove PNP from aqueous solutions.

## Research ethics statement

I declare that the current submitted article is ethical and did not use any human or human tissues. Furthermore, I was not required to complete any ethical assessment prior to conducting my research.

## Animal ethics statement

I declare that the current submitted article did not use animals in the study.

## Fieldwork statement

I declare that the current study was conducted and carried out in a chemical laboratory only, and did not include any fieldwork studies.

## Conflicts of interest

There are no conflicts of interest to declare.

## Supplementary Material
